# Assessing laboratory performance for markers of kidney injury/function: a retrospective study based on External Quality Assessment

**DOI:** 10.1590/2175-8239-JBN-2025-0364en

**Published:** 2026-08-03

**Authors:** José Antonio Tesser Poloni, Adriana Vieira, Rafael Monsores Lopes, Diogo Jerônimo, Bruno Santos, Gisele Meinerz, Adagmar Andriolo

**Affiliations:** 1 Controllab, Rio de Janeiro, RJ, Brazil.; 2 Universidade Federal de Ciências da Saúde de Porto Alegre, Porto Alegre, RS, Brazil.; 3 Universidade Federal de São Paulo, Escola Paulista de Medicina, São Paulo, SP, Brazil.; 4 Sociedade Brasileira de Patologia Clínica e Medicina Laboratorial, Rio de Janeiro, RJ, Brazil.

**Keywords:** External Quality Assessment, Kidney Biomarkers, Creatinine, Urinary Albumin, Urinary Total Protein, Laboratory Performance

## Abstract

**Background:**

Chronic kidney disease (CKD) and acute kidney injury (AKI) affect over 850 million individuals worldwide, with diagnosis and management heavily dependent on laboratory biomarkers. Reliable assessment of kidney biomarkers is therefore essential. External Quality Assessment (EQA) plays a critical role in ensuring the accuracy and comparability of results across laboratories.

**Methods:**

We retrospectively analyzed EQA data from a Brazilian proficiency testing provider (January 2009–March 2024). Reported results for serum creatinine, urinary creatinine, urinary albumin, and total urinary proteins were evaluated. Coefficients of variation (CVs) and adequacy percentages (%Adequacy) were compared across methods using the Kruskal–Wallis test. Trends were assessed with the Mann–Kendall test.

**Results:**

For serum and urinary creatinine, amidinohydrolase/oxidase methods consistently showed the lowest CVs (~3–4.5%) compared with Jaffé-based methods. For urinary albumin, turbidimetry demonstrated superior performance (~5.0% CV), while for total urinary proteins, benzethonium chloride methods yielded the best results (~5.0% CV). Across all biomarkers, CVs decreased and %Adequacy increased significantly over the 15-year period, particularly at clinically relevant concentrations. Nevertheless, Jaffé-based creatinine methods remained predominant despite their well-documented specificity limitations.

**Conclusion:**

Over 15 years, participant laboratories improved the precision and adequacy of kidney biomarker measurements in EQA programs. However, persistent reliance on outdated Jaffé-based creatinine methods compromises standardization and clinical reliability. Adoption of more specific methods and participation in clinically relevant EQA programs are essential to further strengthen laboratory quality and patient safety.

## INTRODUCTION

Chronic kidney disease (CKD) affects an estimated 700 million individuals globally. When the burden of acute kidney injury (AKI), kidney failure, dialysis, and kidney transplantation is included, the global prevalence rises to approximately 850 million people—more than 10% of the world population^1,2,3^. This estimate is likely conservative due to the lack of early detection and screening programs in many regions, which contributes to widespread underdiagnosis, particularly in the early stages of CKD^1,4^. In 2025, the WHO classified CKD as a global health care priority, emphasizing the need for prevention, early detection, and management of kidney disease^5^. AKI is a major contributor to kidney-related morbidity, affecting 7–18% of hospitalized patients and occurring in 20–200 individuals per million annually in community settings^1,6^. The burden is disproportionately higher in resource-constrained settings, where up to 75% of AKI cases are community-acquired and often linked to infections, environmental toxins, and pregnancy-related complications^1,7,8^.

The diagnosis of AKI commonly relies on the KDIGO (Kidney Disease: Improving Global Outcomes) criteria, which include (i) an increase in serum creatinine by ≥0.3 mg/dL (≥26.5 μmol/L) within 48 hours; (ii) an increase in serum creatinine to ≥1.5 times the baseline within the prior seven days; or (iii) a urine output >0.5 mL/kg/h for at least six hours^9,10^.

For CKD, the KDIGO definition includes abnormalities in kidney structure or function lasting at least three months, with health implications, and classifies CKD according to the CGA system—Cause, GFR category (G1–G5), and Albuminuria category (A1–A3)—each of which is critical for assessing disease severity and risk^11^. In clinical practice, GFR is usually estimated using creatinine-based equations; therefore, the reliability of the test is essential for correct diagnosis and management^11^.

Among the key laboratory markers used for diagnosing and monitoring kidney injury and function are serum creatinine, urinary creatinine, urinary albumin, and total urinary protein^12^. Modern clinical laboratories play a central role in delivering these test results, which directly inform medical decision-making^13^. To ensure the reliability and consistency of these results, External Quality Assessment Programs (EQAP) serve as a fundamental component of quality management, allowing laboratories to evaluate all phases of the testing process, including analytical performance, interpretive reporting, and decision thresholds^14^.

KDIGO recommends that serum creatinine testing should be consistent, standardized, and comparable between laboratories, and when possible, should be paired with cystatin C measurement. Globally, most creatinine measurements are performed using colorimetric assays based on the Jaffé reaction, which also react with a variety of non-creatinine substances (chromogens, such as glucose, aspirin, and lipids). Enzymatic methods are more specific for creatinine and less prone to interference, although they are not immune to it (e.g., bilirubin and N-acetylcysteine)^11^. The aspects of measurement error that laboratories need to manage include accuracy (the closeness of the result to the true value), imprecision (analytical variability of the result, usually expressed as the coefficient of variation [CV]), and specificity (the minimization of interferences that may affect the measurement).

Given the global importance of kidney disease and the clinical reliance on laboratory-based assessments, this study aims to evaluate the performance of laboratory methods over time on traditional markers of kidney injury and function using EQA data.

## METHODS

A retrospective analysis was conducted using the database of a Brazilian proficiency testing provider accredited according to ABNT-NBR-ISO/IEC 17043:2011, covering the period from January 2009 to March 2024. The study included results reported by laboratories participating in External Quality Assessment (EQA) programs for serum creatinine, urinary creatinine, urinary albumin, and total urinary proteins.

EQA programs, also referred to as proficiency testing schemes, are designed to evaluate the analytical performance of clinical laboratories by means of interlaboratory comparison. In these programs, standardized samples are distributed to participating laboratories, which analyze them using their routine methods and report the results to the program provider. The results are then compared with assigned or consensus values, allowing the identification of systematic or random errors, the assessment of analytical accuracy, and the promotion of continuous quality improvement.

EQA control materials distributed in four rounds per year were produced using fresh-frozen, lyophilized serum (creatinine) and urine (creatinine, albumin, and total protein), both of which were minimally manipulated.

Coefficients of variation (CVs) and adequacy percentages (%Adequacy) were evaluated over time to identify performance trends and differences among analytical methods. CVs and %Adequacy are commonly used metrics in EQA reports. The CV expresses the analytical imprecision of a set of results, defined as the ratio of the standard deviation to the mean, and is useful for comparing variability among laboratories or methods. The %Adequacy, in turn, reflects the proportion of results that fall within acceptable performance limits established by the EQA provider, serving as an indicator of overall analytical accuracy and compliance with quality specifications. The Kruskal–Wallis test was used to compare CVs across different methods. The Mann–Kendall trend test was applied to assess positive or negative trends in CVs and %Adequacy over time.

To minimize the influence of highly dispersed groups and facilitate the visual interpretation of the data, the Maximum Permissible Dispersion (MPD) from the most recent round of each assay during the evaluated period was used as a cutoff criterion to exclude excessively dispersed results from the boxplot representations.

## RESULTS

**Figure 1:**
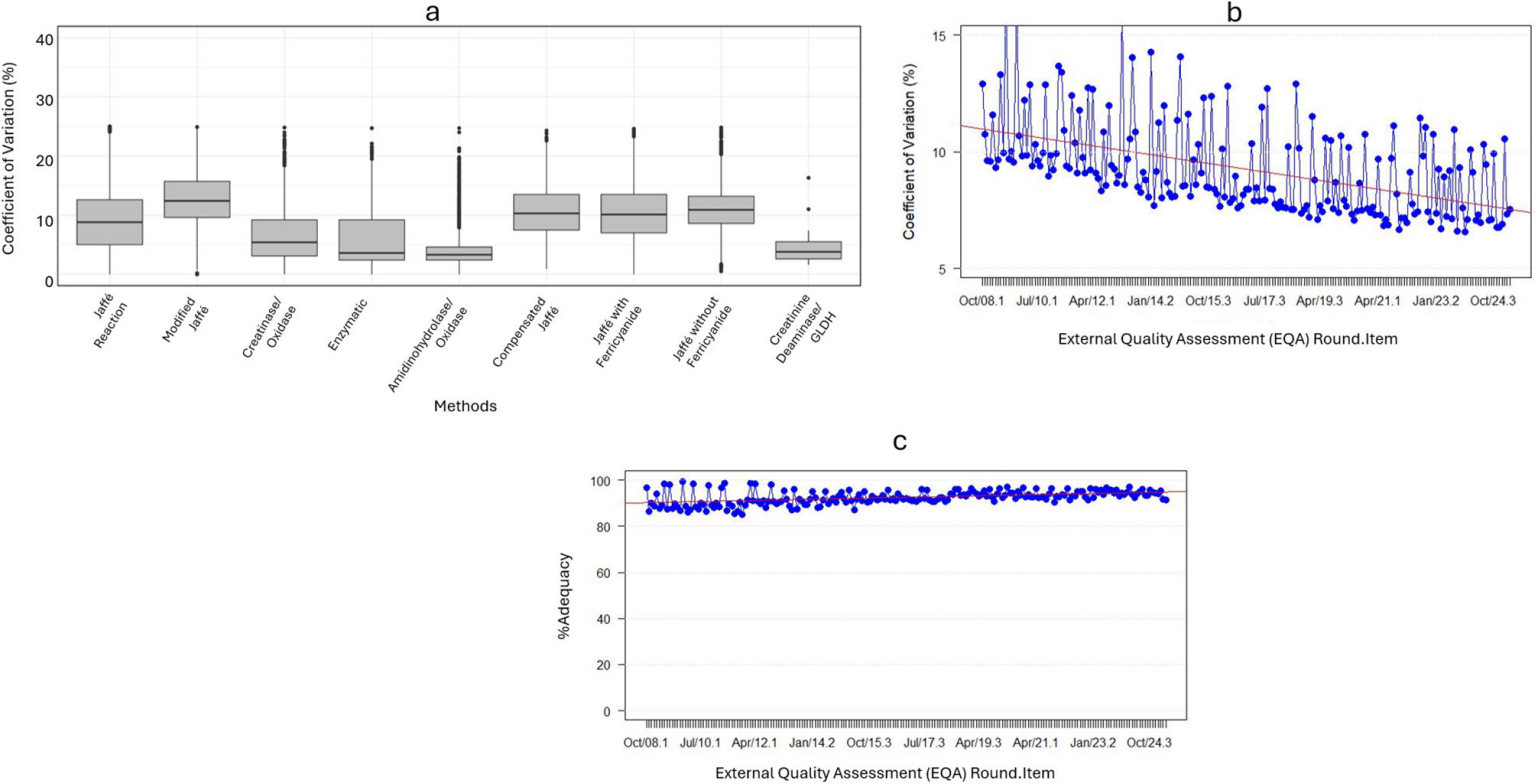
Serum creatinine (all concentrations) a: Methods vs. Coefficient of Variation (CV) (p < 0.0001) b: Trends in Coefficient of Variation (CV) (p < 0.0001) c: Trends in %Adequacy (p < 0.0001).

Serum creatinine measurements included 4,927 laboratories (4,501 from Brazil), totaling 303,983 datasets (MPD for analysis: 25%). The most frequently reported methods were the Jaffé-based (N = 197,597), modified Jaffé (N = 29,271), those based on amidinohydrolase/oxidase (N = 28,951), and other methods (N = 48,164). The most frequent concentrations in EQA rounds were above 2 mg/dL, and at these levels, coefficients of variation (CVs) tended to be lower, indicating less dispersed peer groups and better analytical performance. Significant differences in CVs among methods were observed regardless of concentration (p < 0.0001) (Figure 1a), with the best median performance observed for enzymatic (~3.4%) and amidinohydrolase/oxidase (~3.3%) methods. Over time, a consistent decreasing trend in CVs (p < 0.0001) (Figure 1b) and an increasing trend in %Adequacy across all concentrations (p < 0.0001) (Figure 1c) were observed.

**Figure 2:**
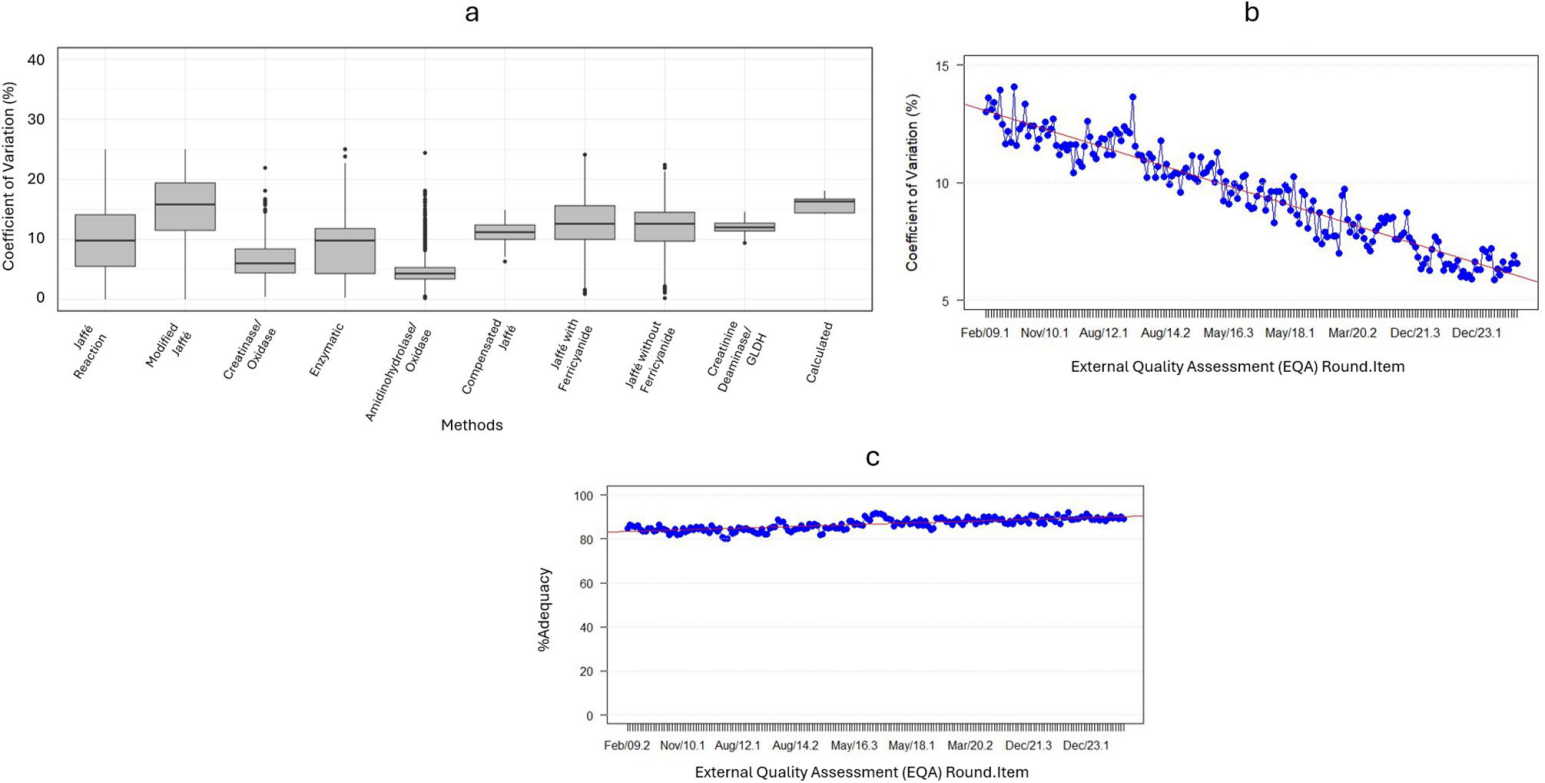
Urinary creatinine (all concentrations) a: Methods vs. Coefficient of Variation (CV) (p < 0.0001) b: Trends in Coefficient of Variation (CV) (p < 0.0001) c: Trends in %Adequacy (p < 0.0001).

Urinary creatinine measurements comprised 1,936 laboratories (1,887 from Brazil), totaling 125,805 datasets (MPD for analysis: 25%). Jaffé-based methods were again the most frequent (N = 87,129), followed by methods based on amidinohydrolase/oxidase (N = 14,040), the ferricyanide Jaffé reaction (N = 7,457), and other methods (N = 17,179). The most common concentrations ranged from 500 to 1,000 mg/L, without clear CV variation according to concentration within each method. However, significant differences between methods were observed at all concentrations (p < 0.0001) (Figure 2a), with methods based on amidinohydrolase/oxidase showing the best median performance (~4.5%). A significant temporal decrease in CVs (p < 0.0001) (Figure 2b) and an increase in %Adequacy (p < 0.0001) (Figure 2c) were also found.

**Figure 3:**
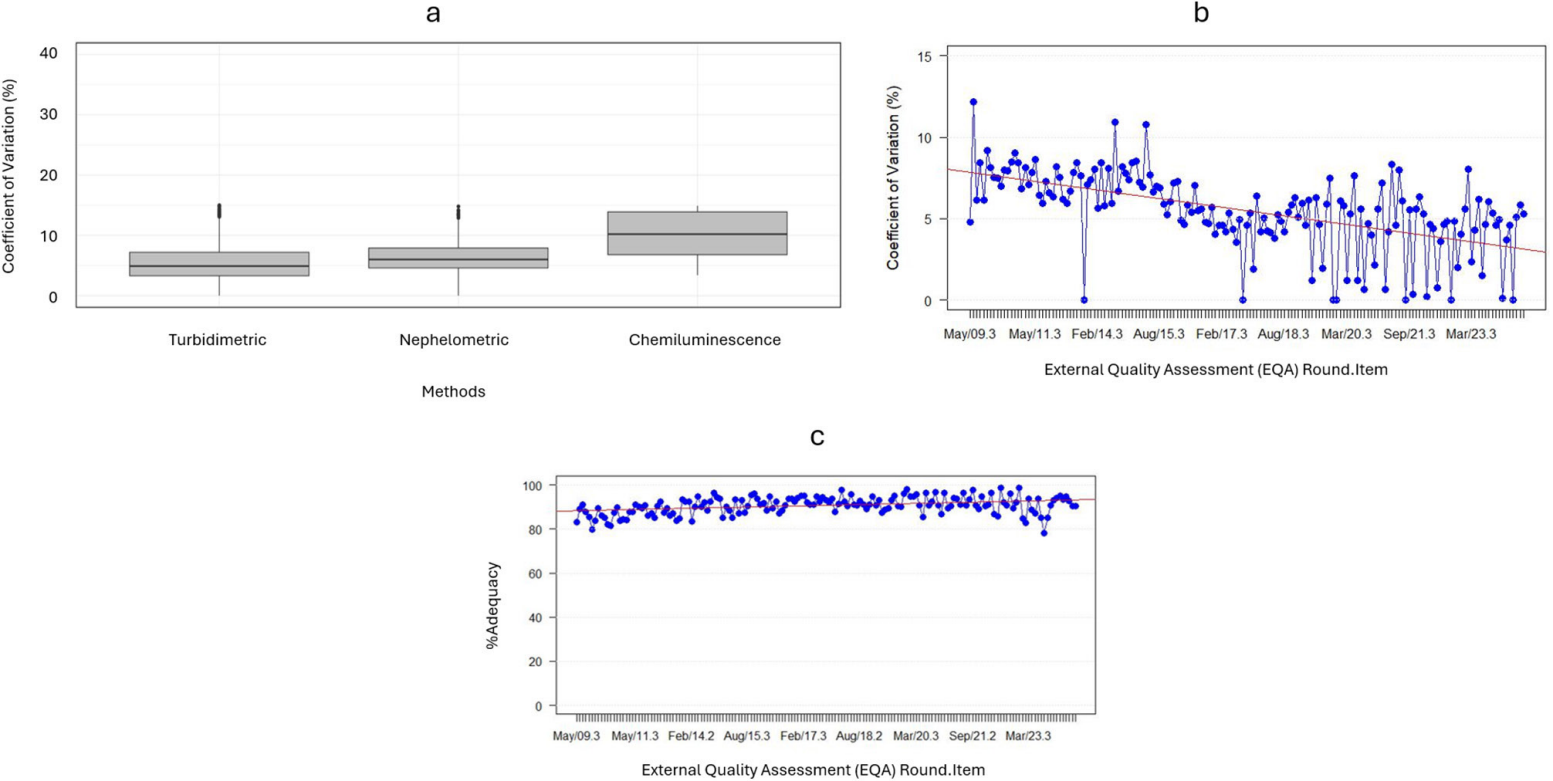
Urinary albumin (all concentrations) a: Methods vs. Coefficient of Variation (CV) (p < 0.0001) b: Trends in Coefficient of Variation (CV) (p < 0.0001) c: Trends in %Adequacy (p < 0.0001).

For urinary albumin, 570 laboratories (543 from Brazil) contributed with 21,150 datasets (MPD for analysis: 15%). Turbidimetric (N = 18,799) and nephelometric (N = 2,201) methods predominated, while chemiluminescence was reported by 150 participants. The most frequent concentrations ranged from 30 to 300 mg/L, with no clear concentration-related CV changes within method groups. Significant differences among methods were observed at all concentrations (p < 0.0001) (Figure 3a), and the best median performance was achieved by the turbidimetric method (~5.0%). Over the study period, CVs showed a significant decreasing trend (p < 0.0001) (Figure 3b), while %Adequacy showed a significant increase (p < 0.0001) across all concentrations (Figure 3c).

**Figure 4:**
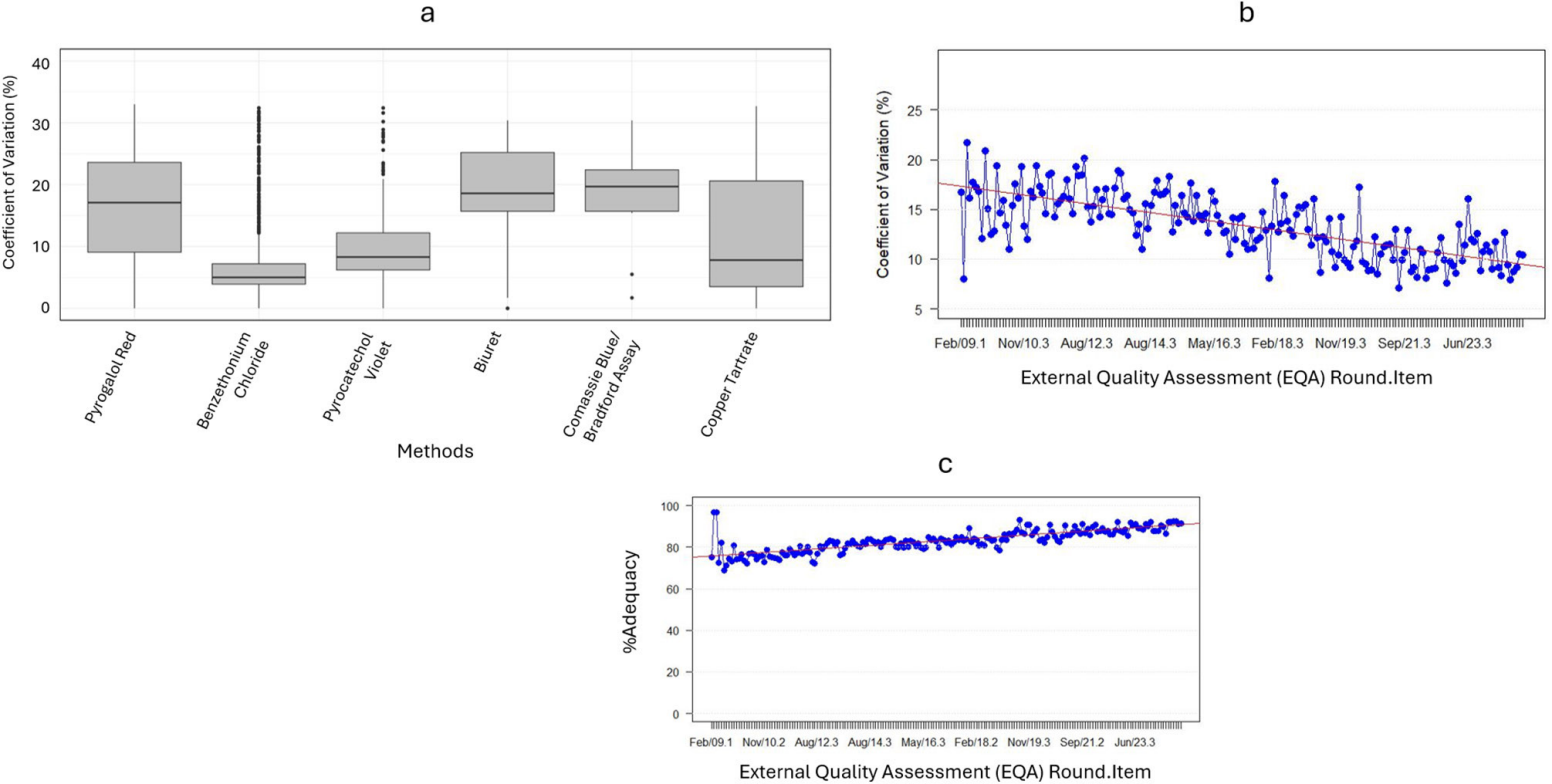
Urinary total protein (all concentrations) a: Methods vs. Coefficient of Variation (CV) (p < 0.0001) b: Trends in Coefficient of Variation (CV) (p < 0.0001) c: Trends in %Adequacy (p < 0.0001).

Total urinary protein measurements included 1,748 laboratories (1,706 from Brazil), corresponding to 89,470 datasets (MPD for analysis: 33%). The most frequently reported methods were pyrogallol red (N = 58,512), benzethonium chloride (N = 20,286), pyrocatechol violet (N = 8,861), and other methods (N = 1,811). The most common concentrations ranged from 0.5 to 1.0 g/L, with no clear CV variation by concentration. Significant differences between methods were observed at all concentrations (p < 0.0001) (Figure 4a), and benzethonium chloride showed the best median performance (~5.0%). Significant decreasing trends in CVs (p < 0.0001) (Figure 4b) and increasing trends in %Adequacy (p < 0.0001) (Figure 4c) were observed over the rounds.

## DISCUSSION

Serum and urinary creatinine, urinary albumin, and total urinary protein are key biomarkers for the screening, classification, and risk assessment of kidney disease. The use of fixed decision thresholds from clinical guidelines requires that different measurement procedures yield equivalent results. Without standardization, misclassification and inappropriate clinical management may occur^15^. EQA programs are central to assessing laboratory performance through interlaboratory comparisons, supporting method standardization, harmonization of results, and compliance with ISO/IEC 15189 requirements^16^. To our knowledge, this is the first long-term study evaluating EQA data for kidney injury/function markers from a Brazilian proficiency testing provider.

For both serum and urinary creatinine, Jaffé-based methods were the most frequently reported, despite their well-documented specificity limitations^13^. In serum creatinine, these assays may negatively affect internal quality control, EQA performance, and patient results, particularly at low concentrations relevant to acute kidney injury algorithms. In both control material matrices (serum and urine), amidinohydrolase/oxidase methods demonstrated the lowest CVs, suggesting superior analytical performance and indicating that broader adoption of these methods may reduce the risk of error. Although the literature on urinary creatinine performance in EQA remains limited^17^, our findings consistently showed progressive improvements in both CVs and %Adequacy over time. Participation in EQA programs has been associated with better laboratory performance through the identification of systematic errors, equipment-related issues, and continuous monitoring, in addition to being a requirement for accreditation^18^. It is noteworthy that Jaffé-based methods remain the most widely used among participating laboratories, despite the availability of more accurate alternatives with well-documented performance. Our findings highlight this persistent reliance on less specific methods and underscore the need for broader efforts to encourage their replacement in routine practice. Although the factors sustaining the continued high use of Jaffé-based assays remain speculative, cost is likely to be an important driver.

Urinary albumin was mainly analyzed by immuno­turbidimetry, which yielded the lowest CV in our study. However, significant inter-platform differences have been reported^19,20^, with important implications for albuminuria classification using the albumin-to-creatinine ratio. These findings reinforce the need for continuous interassay comparison and harmonization efforts. Urinary total protein, although absent from the KDIGO AKI and CKD guidelines, remains widely used in Brazil. In our dataset, benzethonium chloride methods showed the best performance, although the literature addressing EQA-based performance for this analyte is still scarce and outdated^21^. Encouragingly, analytical performance improved consistently across the study period.

Overall, this 15-year dataset from a large Brazilian program provides rare insight into longitudinal trends in laboratory performance for kidney biomarkers. Precision and adequacy improved for all biomarkers over time; however, the persistent use of less specific creatinine methods remains a relevant concern for standardization and clinical reliability. These findings emphasize the need for continued harmonization of kidney biomarker measurements to improve comparability across laboratories and support reliable clinical decision-making. Laboratories should prioritize EQA programs that provide clinically relevant test items and robust assessments of assay performance^13^.

The main limitations of this study include the use of fresh-frozen, lyophilized, minimally manipulated human serum and urine controls, for which commutability was not formally assessed, as well as the absence of clinical data and other potential confounding factors inherent to the study design. Its main strengths are the large number of measurements and the long-term longitudinal analysis. Over 15 years, participating laboratories improved the precision and adequacy of results for kidney injury/function biomarkers. Amidinohydrolase/oxidase methods for serum and urinary creatinine, immunoturbidimetry for urinary albumin, and benzethonium chloride methods for urinary total protein demonstrated superior performance. However, the continued reliance on Jaffé-based creatinine methods may still compromise standardization and clinical reliability. Broader adoption of more specific methods, coupled with participation in challenging and clinically relevant EQA programs, is essential to further enhance laboratory quality and patient care.

## Data Availability

The datasets generated and/or analyzed during the current study are not publicly available due to ethical, legal, and/or privacy restrictions but are available from the corresponding author upon reasonable request.
