# Supplementary material for: Assessing laboratory performance for markers of kidney injury/function: a retrospective study based on External Quality Assessment
Source: J Bras Nefrol. 2026 Aug 3;48(4):e20250364. doi: 10.1590/2175-8239-JBN-2025-0364en (PMC13431443; doi:10.1590/2175-8239-JBN-2025-0364en)
Supplement: Translation

# Translation: Avaliação do desempenho laboratorial em marcadores de lesão/função renal: um estudo retrospectivo baseado em Avaliação Externa de Qualidade

**DOI:** 10.1590/2175-8239-JBN-2025-0364pt

**Authors:** José Antonio Tesser Poloni, Adriana Vieira, Rafael Monsores Lopes, Diogo Jerônimo, Bruno Santos, Gisele Meinerz, Adagmar Andriolo

**Affiliations:** 1 Controllab, Rio de Janeiro, RJ, Brasil.; 2 Universidade Federal de Ciências da Saúde de Porto Alegre, Porto Alegre, RS, Brasil.; 3 Universidade Federal de São Paulo, Escola Paulista de Medicina, São Paulo, SP, Brasil.; 4 Sociedade Brasileira de Patologia Clínica e Medicina Laboratorial, Rio de Janeiro, RJ, Brasil.

**Keywords:** Avaliação Externa da Qualidade, Biomarcadores Renais, Creatinina, Albumina Urinária, Proteína Total Urinária, Desempenho Laboratorial

## Abstract

**Introdução:**

Doença renal crônica (DRC) e lesão renal aguda (LRA) afetam mais de 850 milhões de pessoas no mundo, e seu diagnóstico e monitoramento dependem fortemente de biomarcadores laboratoriais. A Avaliação Externa da Qualidade (AEQ) é fundamental para garantir a acurácia e a comparabilidade dos resultados entre laboratórios.

**Métodos:**

Foram analisados retrospectivamente dados de AEQ de um provedor brasileiro entre janeiro (2009) e março (2024). Avaliaram-se os resultados de creatinina sérica e urinária, albumina urinária e proteínas totais urinárias. Os coeficientes de variação (CVs) e os percentuais de adequação (%Adequação) foram comparados entre métodos pelo teste de *Kruskal–Walli*s, e as tendências temporais foram avaliadas pelo teste de *Mann–Kendall*.

**Resultados:**

Os métodos de creatinina baseados em amidinohidrolase/oxidase apresentaram os menores CVs (aproximadamente 3,0–4,5%), superando consistentemente os métodos de *Jaffé*. Para albumina urinária, a turbidimetria mostrou melhor desempenho (CV ~5,0%), enquanto, para proteínas totais urinárias, os métodos baseados em cloreto de benzetônio apresentaram os melhores resultados (CV ~5,0%). Em todos os biomarcadores, observou-se redução significativa dos CVs e aumento do %Adequação ao longo dos 15 anos, especialmente em concentrações clinicamente relevantes. Apesar disso, os métodos de creatinina baseados em *Jaffé* permaneceram os mais utilizados.

**Conclusão:**

Os laboratórios participantes apresentaram melhora significativa na precisão e adequação das medições de biomarcadores renais ao longo de 15 anos de AEQ. Entretanto, a persistente utilização de métodos de *Jaffé* limita a padronização e a confiabilidade clínica, reforçando a necessidade da adoção de métodos mais específicos e da participação contínua em programas de AEQ.

## INTRODUÇÃO

A doença renal crônica (DRC) afeta aproximadamente 700 milhões de indivíduos globalmente. Quando considerada também a carga de lesão renal aguda (LRA), falência renal, diálise e transplante renal, a prevalência global aumenta para aproximadamente 850 milhões de pessoas—mais de 10% da população mundial^1,2,3^. Essa estimativa é provavelmente conservadora devido à ausência de programas de detecção precoce e rastreamento em muitas regiões, o que contribui para o subdiagnóstico generalizado, particularmente nos estágios iniciais da DRC^1,4^. Em 2025, a OMS classificou a DRC como prioridade em saúde, enfatizando a necessidade de prevenção, detecção precoce e manejo da doença renal^5^.

A LRA é um importante fator de morbidade, afetando de 7% a 18% dos pacientes hospitalizados e ocorrendo em 20 a 200 indivíduos por milhão, anualmente, em ambientes comunitários^1,6^. A carga é desproporcionalmente maior em cenários com recursos limitados, onde até 75% dos casos de LRA são adquiridos na comunidade e, frequentemente, relacionados a infecções, toxinas ambientais e complicações da gravidez^1,7,8^.

O diagnóstico de LRA se baseia comumente nos critérios do KDIGO *(Kidney Disease: Improving Global Outcomes*), que incluem: (i) aumento da creatinina sérica ≥0,3 mg/dL (≥26,5 μmol/L) em 48 horas; (ii) aumento da creatinina sérica para ≥1,5 vezes o valor basal nos sete dias anteriores; ou (iii) débito urinário >0,5 mL/kg/h por pelo menos seis horas^9,10^.

Para a DRC, a definição do KDIGO inclui anormalidades na estrutura ou na função renal com duração mínima de três meses e implicações para a saúde. A DRC é classificada pelo sistema CGA—Causa, categoria de TFG (G1–G5) e categoria de albuminúria (A1–A3)—, sendo cada componente crítico para a avaliação da gravidade e do risco^11^. Na prática clínica, a TFG é usualmente estimada por equações baseadas em creatinina, tornando a confiabilidade do teste essencial para o diagnóstico e o manejo corretos^11^.

Entre os principais marcadores laboratoriais utilizados no diagnóstico e no monitoramento da lesão e da função renais estão creatinina sérica, creatinina urinária, albumina urinária e proteína total urinária^12^. Os laboratórios clínicos modernos desempenham papel central na produção desses resultados, que impactam diretamente a tomada de decisão médica^13^. Para assegurar a confiabilidade e a consistência desses resultados, os Programas de Avaliação Externa da Qualidade (PAEQ) constituem um componente fundamental da gestão da qualidade, permitindo aos laboratórios avaliar todas as fases do processo analítico, incluindo desempenho analítico, interpretação de resultados e limites de decisão^14^.

O KDIGO recomenda que a dosagem de creatinina sérica seja consistente, padronizada e comparável entre laboratórios e, quando possível, associada à medição de cistatina C. Globalmente, a determinação da creatinina é realizada predominantemente por ensaios colorimétricos baseados na reação de *Jaffé*, os quais também reagem com diversas substâncias além da creatinina (cromógenos, como glicose, aspirina e lipídios). Métodos enzimáticos são mais específicos para a dosagem de creatinina e menos suscetíveis a interferências, embora não sejam imunes a elas (por exemplo, bilirrubina e N-acetilcisteína)^11^. Os aspectos do erro de medição que os laboratórios precisam gerenciar incluem exatidão (a proximidade do resultado com o valor real), imprecisão (variabilidade analítica, geralmente expressa como coeficiente de variação – CV) e especificidade (minimização de interferências que possam alterar a medição).

Dada a importância global da doença renal e a dependência clínica das avaliações laboratoriais, este estudo tem como objetivo avaliar, ao longo do tempo, o desempenho dos métodos laboratoriais na determinação de marcadores tradicionais de lesão e função renais utilizando dados de AEQ.

## MÉTODOS

Foi realizada uma análise retrospectiva utilizando o banco de dados de um provedor brasileiro de ensaios de proficiência acreditado de acordo com a ABNT-NBR-ISO/IEC 17043:2011, abrangendo o período de janeiro de 2009 a março de 2024. O estudo incluiu resultados relatados por laboratórios participantes de programas de Avaliação Externa da Qualidade (AEQ) para creatinina sérica, creatinina urinária, albumina urinária e proteínas totais urinárias.

Os programas de AEQ, também denominados programas de ensaio de proficiência, são projetados para avaliar o desempenho analítico de laboratórios clínicos por meio de comparação interlaboratorial. Nesses programas, amostras padronizadas são distribuídas aos laboratórios participantes, que as analisam utilizando seus métodos de rotina e relatam os resultados ao provedor do programa. Os resultados são então comparados com valores atribuídos ou consensuais, permitindo a identificação de erros sistemáticos ou aleatórios, a verificação da precisão analítica e a promoção da melhoria contínua da qualidade.

Os materiais de controle da AEQ enviados em quatro rodadas anuais foram produzidos utilizando soro liofilizado fresco congelado (creatinina) e urina (creatinina, albumina e proteínas totais), ambos minimamente manipulados.

Os coeficientes de variação (CVs) e os percentuais de adequação (%Adequação) foram avaliados ao longo do tempo para identificar tendências de desempenho e diferenças entre os métodos analíticos. CV e %Adequação são métricas comumente utilizadas em relatórios da AEQ. O CV expressa a imprecisão analítica de um conjunto de resultados, representando a razão entre o desvio padrão e a média e sendo útil para comparar a variabilidade entre laboratórios ou métodos. O %Adequação, por sua vez, reflete a proporção de resultados que se enquadram nos limites de desempenho aceitáveis estabelecidos pelo provedor da AEQ, servindo como indicador da exatidão analítica geral e da conformidade com as especificações de qualidade. O teste de *Kruskal–Wallis* foi utilizado para comparar os CVs entre os diferentes métodos. O teste de tendência de *Mann–Kendall* foi aplicado para avaliar tendências positivas ou negativas nos CVs e no %Adequação ao longo do tempo.

Para minimizar a influência de grupos altamente dispersos e facilitar a interpretação visual dos dados, a Dispersão Máxima Permissível (DMP) da rodada mais recente de cada ensaio durante o período avaliado foi utilizada como critério de corte para excluir resultados excessivamente dispersos das representações em *boxplots*.

## RESULTADOS

A análise da creatinina sérica incluiu 4.927 laboratórios (4.501 do Brasil), totalizando 303.983 conjuntos de dados (DMP para análise: 25%). Os métodos mais frequentemente relatados foram os baseados na reação de *Jaffé* (N = 197.597), *Jaffé* modificado (N = 29.271), aqueles baseados em amidinohidrolase/oxidase (N = 28.951) e outros métodos (N = 48.164). As concentrações mais frequentes nas rodadas de AEQ foram acima de 2 mg/dL e, nesses níveis, os CVs tenderam a ser menores, indicando grupos de pares menos dispersos e melhor desempenho analítico. Diferenças significativas nos CVs entre os métodos foram observadas independentemente da concentração (p < 0,0001) (Figura 1a), com o melhor desempenho mediano observado para os métodos enzimáticos (~3,4%) e os métodos baseados em amidinohidrolase/oxidase (~3,3%). Ao longo do tempo, observou-se uma tendência decrescente consistente nos CVs (p < 0,0001) (Figura 1b) e uma tendência crescente do %Adequação em todas as concentrações (p < 0,0001) (Figura 1c).

**Figura 1:** Creatinina sérica (todas as concentrações) a: Métodos vs. Coeficientes de Variação (CVs) (p < 0,0001) b: Tendências nos Coeficientes de Variação (CVs) (p < 0,0001) c: Tendências nos %Adequação (p < 0,0001).

A medição da creatinina urinária compreendeu 1.936 laboratórios (1.887 do Brasil), totalizando 125.805 conjuntos de dados (DMP para análise: 25%). Os métodos baseados na reação de *Jaffé* foram novamente os mais frequentes (N = 87.129), seguidos por métodos baseados em amidinohidrolase/oxidase (N = 14.040), reação de *Jaffé* com ferricianeto (N = 7.457) e outros métodos (N = 17.179). As concentrações mais comuns variaram de 500 a 1.000 mg/L, sem variação clara do CV de acordo com a concentração dentro de cada método. No entanto, diferenças significativas entre os métodos foram observadas em todas as concentrações (p < 0,0001) (Figura 2a), com os métodos baseados em amidinohidrolase/oxidase apresentando o melhor desempenho mediano (~4,5%). Observou-se também redução temporal significativa nos CVs (p < 0,0001) (Figura 2b) e aumento no %Adequação (p < 0,0001) (Figura 2c).

**Figura 2:** Creatinina urinária (todas as concentrações) a: Métodos vs. Coeficientes de Variação (CVs) (p < 0,0001) b: Tendências nos Coeficientes de Variação (CVs) (p < 0,0001) c: Tendências nos %Adequação (p < 0,0001).

Para albumina urinária, 570 laboratórios (543 do Brasil) contribuíram com 21.150 conjuntos de dados (DMP para análise: 15%). Os métodos turbidimétricos (N = 18.799) e nefelométricos (N = 2.201) predominaram, enquanto a quimioluminescência foi relatada por 150 participantes. As concentrações mais frequentes variaram de 30 a 300 mg/L, sem alterações claras nos CVs relacionadas à concentração dentro de cada grupo de métodos. Diferenças significativas entre os métodos foram observadas em todas as concentrações (p < 0,0001) (Figura 3a), e o melhor desempenho mediano foi alcançado pelo método turbidimétrico (~5,0%). Ao longo do período do estudo, os CVs mostraram uma tendência decrescente significativa (p < 0,0001) (Figura 3b), enquanto o %Adequação apresentou aumento significativo (p < 0,0001) em todas as concentrações (Figura 3c).

**Figura 3:** Albumina urinária (todas as concentrações) a: Métodos vs. Coeficientes de Variação (CVs) (p < 0,0001) b: Tendências nos Coeficientes de Variação (CVs) (p < 0,0001) c: Tendências nos %Adequação (p < 0,0001).

As determinações de proteínas totais urinárias incluíram 1.748 laboratórios (1.706 do Brasil), correspondendo a 89.470 conjuntos de dados (DMP para análise: 33%). Os métodos mais frequentemente relatados foram vermelho de pirogalol (N = 58.512), cloreto de benzetônio (N = 20.286), violeta de pirocatecol (N = 8.861) e outros métodos (N = 1.811). As concentrações mais comuns variaram de 0,5 a 1,0 g/L, sem variação clara do CV de acordo com a concentração. Diferenças significativas entre os métodos foram observadas em todas as concentrações (p < 0,0001) (Figura 4a), e o método baseado em cloreto de benzetônio apresentou o melhor desempenho mediano (~5,0%). Tendências significativas de redução dos CVs (p < 0,0001) (Figura 4b) e de aumento do %Adequação (p < 0,0001) (Figura 4c) foram observadas ao longo das rodadas.

**Figura 4:** Proteínas totais urinárias (todas as concentrações) a: Métodos vs. Coeficientes de Variação (CVs) (p < 0,0001) b: Tendências nos Coeficientes de Variação (CVs) (p < 0,0001) c: Tendências nos %Adequação (p < 0,0001).

## DISCUSSÃO

A creatinina sérica e urinária, a albumina urinária e as proteínas totais urinárias são biomarcadores essenciais para o rastreio, a classificação e a estratificação de risco de doença renal. A utilização de limiares de decisão fixos, conforme diretrizes clínicas, exige que diferentes procedimentos de medição produzam resultados equivalentes. Sem padronização, podem ocorrer classificações incorretas e manejo clínico inadequado^15^. Os Programas de Avaliação Externa da Qualidade (PAEQs) são fundamentais para avaliar o desempenho laboratorial por meio de comparações interlaboratoriais, apoiando a padronização de métodos, a harmonização de resultados e a conformidade com os requisitos da ISO/IEC 15189^16^. Até onde sabemos, este é o primeiro estudo de longo prazo a avaliar dados de PAEQ relativos a marcadores de lesão/função renal obtidos de um provedor brasileiro de ensaios de proficiência.

Tanto para a creatinina sérica quanto para a urinária, os métodos baseados na reação de *Jaffé* foram os mais frequentemente relatados, apesar de suas limitações de especificidade bem documentadas^13^. Na creatinina sérica, esses ensaios podem afetar negativamente o controle interno da qualidade, o desempenho em PAEQs e os resultados dos pacientes, particularmente em baixas concentrações relevantes para algoritmos de lesão renal aguda. Em ambas as matrizes de material de controle (soro e urina), os métodos de amidinohidrolase/oxidase demonstraram os menores CVs, sugerindo um desempenho analítico superior e indicando que uma adoção mais ampla desses métodos pode reduzir o risco de erros. Embora a literatura sobre o desempenho da creatinina urinária em PAEQs ainda seja limitada^17^, nossos resultados demonstraram consistentemente melhorias progressivas tanto nos CVs quanto no %Adequação ao longo do tempo. A participação em PAEQs tem sido associada a um melhor desempenho laboratorial por meio da identificação de erros sistemáticos, de problemas relacionados a equipamentos e do monitoramento contínuo, além de ser um requisito para acreditação^18^. É importante destacar que os métodos baseados na reação de *Jaffé* continuam sendo os mais utilizados entre os laboratórios participantes, apesar da disponibilidade de alternativas mais precisas com desempenho bem documentado. Nossos resultados destacam essa dependência persistente de métodos menos específicos e ressaltam a necessidade de esforços mais amplos para incentivar sua substituição nas práticas de rotina. Embora os fatores que sustentam a elevada utilização de ensaios baseados na reação de *Jaffé* permaneçam especulativos, o custo provavelmente desempenhe um papel determinante.

A albumina urinária foi analisada principalmente por imunoturbidimetria, que apresentou o menor CV em nosso estudo. No entanto, diferenças significativas entre plataformas foram relatadas^19,20^, com implicações importantes para a classificação da albuminúria utilizando a razão albumina/creatinina. Esses achados reforçam a necessidade de comparações interensaio contínuas e de esforços de harmonização. As proteínas totais urinárias, embora ausentes das diretrizes KDIGO para LRA e DRC, permanecem amplamente utilizadas no Brasil. Em nosso conjunto de dados, os métodos baseados em cloreto de benzetônio apresentaram os melhores resultados, embora a literatura sobre o desempenho desse analito em programas de AEQ ainda seja escassa e desatualizada^21^. De maneira encorajadora, o desempenho analítico melhorou consistentemente ao longo do período do estudo.

No geral, este conjunto de dados de 15 anos de um grande programa brasileiro fornece evidências raras sobre as tendências longitudinais do desempenho laboratorial de biomarcadores renais. A precisão e a adequação melhoraram para todos os biomarcadores ao longo do tempo; no entanto, o uso persistente de métodos de creatinina menos específicos continua sendo uma preocupação relevante para a padronização e a confiabilidade clínica. Esses achados enfatizam a necessidade de harmonização contínua das medições de biomarcadores renais para melhorar a comparabilidade entre laboratórios e apoiar a tomada de decisões clínicas confiáveis. Os laboratórios devem priorizar programas de AEQ que forneçam itens de teste clinicamente relevantes e avaliações robustas do desempenho do ensaio^13^.

As principais limitações deste estudo incluem o uso de controles de soro e urina humanos frescos congelados e liofilizados minimamente manipulados, para os quais a comutabilidade não foi formalmente avaliada, bem como a ausência de dados clínicos e de outros potenciais fatores de confusão inerentes ao desenho do estudo. Seus principais pontos fortes são o grande número de medições e a análise longitudinal de longo prazo. Ao longo de 15 anos, os laboratórios participantes melhoraram a precisão e a adequação dos resultados para biomarcadores de lesão/função renal. Os métodos de amidinohidrolase/oxidase para creatinina sérica e urinária, a imunoturbidimetria para albumina urinária e os métodos baseados em cloreto de benzetônio para proteínas totais urinárias demonstraram desempenho superior. No entanto, a dependência contínua de métodos de creatinina baseados na reação de *Jaffé* ainda pode comprometer a padronização e a confiabilidade clínica. A adoção mais ampla de métodos mais específicos, juntamente com a participação em PAEQs desafiadores e clinicamente relevantes, é essencial para aprimorar ainda mais a qualidade laboratorial e o atendimento ao paciente.

## Data Availability

Os conjuntos de dados gerados e/ou analisados durante o presente estudo não estão disponíveis publicamente devido a restrições éticas, legais e/ou de privacidade, mas podem ser disponibilizados mediante solicitação razoável ao autor correspondente.
